# BOLDsωimsuite: A new software suite for forward modeling of the BOLD fMRI signal

**DOI:** 10.1162/imag_a_00519

**Published:** 2025-03-31

**Authors:** Jacob Chaussé, Avery J. L. Berman, J. Jean Chen

**Affiliations:** Rotman Research Institute, Baycrest, Toronto, Canada; Department of Chemistry, University of Waterloo, Waterloo, Canada; Department of Physics, Carleton University, Ottawa, Canada; University of Ottawa Institute of Mental Health Research, Ottawa, Canada; Department of Medical Biophysics, University of Toronto, Toronto, Canada; Department of Biomedical Engineering, University of Toronto, Toronto, Canada

**Keywords:** BOLD fMRI, forward modeling, biophysical modelling, static dephasing, diffusion, Monte Carlo simulations, gradient-echo signal, spin-echo signal, quantitative MRI, vascular fingerprinting, calibrated fMRI, iron mapping, Python

## Abstract

Many methods for the forward modeling of the blood-oxygenation level-dependent (BOLD) effect have been created and analyzed to elucidate the mechanisms of BOLD functional MRI (fMRI) techniques and to expand on the potential of the transverse relaxation time (T_2_*) in quantitative MRI. Simulations of this nature can be difficult to implement without prior experience, and differences made by methodological choices can be unclear, which provides a significant barrier of entry into the field. In this paper, we present BOLDsωimsuite, a toolbox for forward modeling of the BOLD effect, which collects many of the principal methods used in the literature into a single coherent package. Implemented as a Python package, simulations are made using scripts by combining various simulation components, thereby providing flexibility in methodological choices. The goal of this toolbox is to provide an open-source, reproducible simulation software suite that is adaptable for different MRI applications, and to which additional features can be added by the user with relative ease. This paper first provides an overview of the methods available in the package and how these methods can be constructed from the toolbox’s modular code components. Then, a brief theoretical explanation of each simulation component is given, supported by the relevant contributors. Next, sample simulations and analyzes that can be created using the package are presented to display its features. Finally, recommendations regarding computational requirements are included to help users choose the best simulation methods to fit their needs. This package has many use cases and significantly reduces methodological barriers to forward modeling. It can also be a good learning tool for MR physics as well as a powerful tool to promote reproducible science.

## 1 Introduction

While the blood-oxygenation level-dependent (BOLD) functional MRI (fMRI) technique has become widely used, the full complexities of its origins remain poorly understood by most users. In addition to being used in studying functional brain organization, the transverse relaxation (*T*_2_*)-weighted signal underlying the BOLD technique has also increasingly been used in quantitative MRI applications such as iron quantification (Kor et al., 2019) and the modeling of contrast agent-induced MR signals (Báez-Yánez et al., 2017;Bieri and Scheffler, 2007;Pannetier et al., 2013). Modeling of the*T*_2_*-weighted BOLD signal not only helps to deepen the understanding of BOLD signal origins, but is also invaluable in consolidating the interpretation and signal changes in the above applications.

Dynamic modeling of the BOLD signal has used empirical models to characterize the dependence of the*T*_2_*-weighted signal evolution on variables such as cerebral blood flow (CBF), arteriolar/venous cerebral blood volume (CBV), blood oxygenation, echo time (TE), and field strength (B_0_) (Markuerkiaga et al., 2016;Uludağ et al., 2009). These modeling approaches, in turn, rely on summary measures that were derived from forward modeling of the BOLD signal from the underlying vasculature, which could exhibit different properties depending on the individual, health condition, and so on. Thus, BOLD forward modeling can enable powerful quantitative MRI approaches, such as vascular MR fingerprinting (Christen et al., 2014), which will be detailed later in this work.

Forward modeling of the BOLD signal involves the use of microscopic physics to simulate bulk BOLD signals arising from the mesoscopic structure of the vasculature. An analytical expression of*T*_2_* as a function of perturber geometry (specifically cylinders), blood oxygenation, and subsequently field offset (Chu et al., 1990) in the context of BOLD fMRI was introduced byOgawa et al. (1993). Early work to model the BOLD signal used the Monte Carlo approach to model water diffusion and treated blood vessels as infinite cylinders (Boxerman, Bandettini, et al., 1995;Kennan et al., 1994;Ogawa et al., 1993). This work demonstrated that the BOLD signal is highly sensitive to vessel size, due to the dynamic dephasing effects of diffusion in the extra-vascular space (Boxerman, Bandettini, et al., 1995;Kennan et al., 1994).Yablonskiy and Haacke (1994)established physical analytical models of BOLD contrast based on the case of static dephasing that could predict the gradient-echo BOLD effect around intermediate to large vessels, but still not capturing the BOLD signal around capillaries. Since then, many approaches to capturing the diffusion sensitivity of the BOLD signal have been introduced, where Monte Carlo-based methods have become established as the mainstream for diffusion modeling in BOLD fMRI (Boxerman, Bandettini, et al., 1995;Boxerman, Hamberg, et al., 1995;Gagnon et al., 2015;Martindale et al., 2008;Mueller-Bierl et al., 2007;Pathak et al., 2008;Stone et al., 2019). The random-cylinder-based single-voxel predictions have since been validated against those generated from rat vascular-anatomical networks (VAN) (Marques & Bowtell, 2008). Alternate approaches to simulating diffusion have also been proposed, including analytical approximations (Kiselev & Posse, 1999), a deterministic method based on convolution of a diffusion kernel with a magnetization matrix was proposed byBandettini and Wong (1995), and has been successfully applied for various applications (Klassen & Menon, 2007;Pannetier et al., 2014). Among these approaches are two- (2D) and three-dimensional (3D) variants, with the 3D approach being more physically realistic but also entailing more computational costs. These different approaches have different advantages and drawbacks, which will be discussed later. Lastly, BOLD forward modeling has been integrated with neurovascular coupling and multi-voxel simulations for broader utility (Chen & Calhoun, 2011,^2016^;Chen et al., 2013,^2018^;Denk et al., 2011;Fieremans & Lee, 2018;Sukstanskii & Yablonskiy, 2014;Wharton & Bowtell, 2012)

Despite the aforementioned importance of forward modeling for quantitative fMRI, the entry threshold for forward modeling is high compared to that of other approaches, and it is thus no surprise that decades after the inception of BOLD fMRI, quantitative fMRI is still facing low levels of adoption. This is reflected in the low number of publications on the topic of BOLD forward modeling relative to the number of BOLD fMRI researchers. To date, the number of research groups familiar with its implementation remains limited, and the majority of these research groups have not disclosed their codes. Moreover, most of these groups have not used the same simulation approaches, are not actively developing or testing variations on the code, and are instead leveraging legacy code that may not be updated. In an age of open and reproducible science, we find this to be an incongruous knowledge gap. In keeping with the spirit of Open and Reproducible Science,Pannetier et al. (2013)introduced a publicly distributed BOLD modelling toolbox implemented in Matlab, named MrVox. To the best of our knowledge, since its publication, MrVox and its derivative, DCESim, have already been cited in numerous publications and abstracts (Christen et al., 2014;Coudert et al., 2020;Damseh et al., 2021;Delphin et al., 2020,^2021^,^2023^;Lemasson et al., 2016;van Dorth et al., 2022;Venugopal et al., 2023). This not only attests to the need for such a toolbox, but also highlights the importance of validating tools such as MrVox (Berman et al., 2023;Chausse, Berman, & Chen, 2021), which currently implements a single simulation approach (2D Fourier-based deterministic diffusion) in Matlab.

Inspired by this prior work and by the principles of Open and Reproducible Science, and the reception of the prior toolbox by Pannetier et al., we propose BOLDsωimsuite, a comprehensive simulation toolbox implemented entirely in Python that permits Monte Carlo or deterministic diffusion-based simulations with a range of geometries for the sources of magnetic field perturbations in 2D and 3D, using either analytical or Fourier-based field calculations, where applicable. In the following sections, we detail our implementation, their physical principles, highlight some applications, and make recommendations for users.

## 2 Overview of Toolbox

**Fig. 1.**
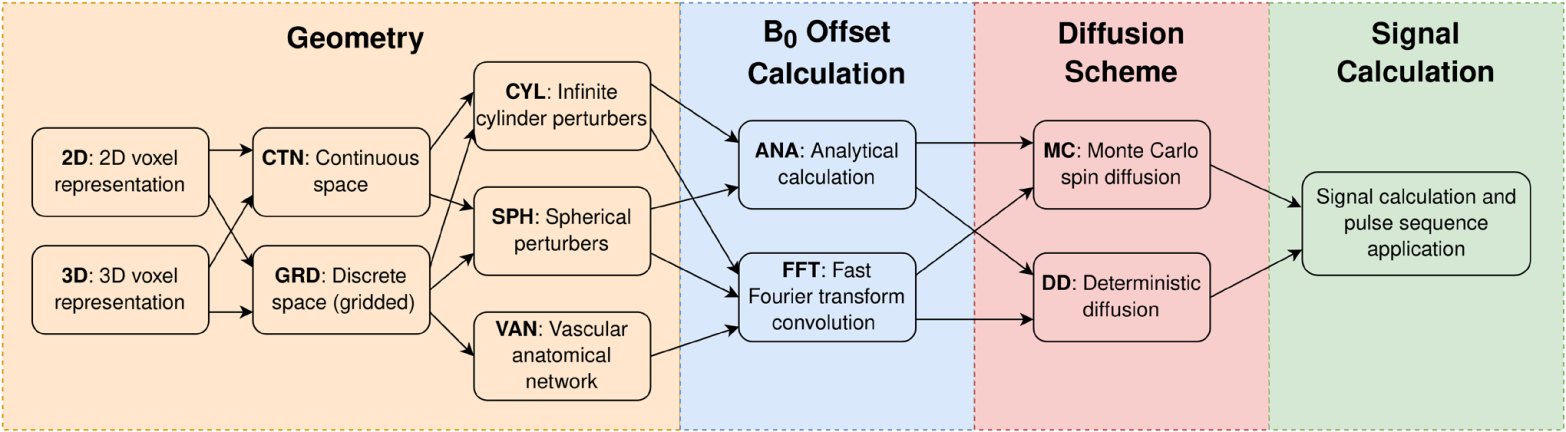
The main components in the numerical simulation pipeline are separated into four categories. Geometry options change the way the vascular network is represented.*B*_0_offset calculation options change the method used to calculate a perturber’s influence on the magnetic field. Diffusion scheme options change how the diffusion of water is represented (either Monte Carlo or deterministic) and how the distribution of magnetization evolves through time. Signal calculation contains many parameters that define the pulse sequence and properties of the signal, such as relaxation times. Each simulation method is given an abbreviated name by combining the bolded short form for each component. For example, a three-dimensional (3D), continuous space (CTN), infinite cylinder (CYL), analytically calculated (ANA), Monte Carlo (MC) simulation would have the abbreviated name of 3D-CTN-CYL-ANA-MC.

The BOLDsωimsuite Toolbox is entirely implemented in Python (Version 3.10). Its various methods are implemented as combinations of methodological choices which are themselves categorized into four components, namely “geometry”, “*B*_0_offset”, “diffusion”, and “signal calculation”.Figure 1shows the four components and their respective options, as well as valid combinations of options. These include

- Geometry options:
  ○ Two- (2D) vs. three-dimensional (3D);
  ○ Continuous (CTN) vs. gridded (GRD) spatial coordinates;
  ○ Cylinder, sphere or custom (VAN);
- *B*_0_offset calculation options:
  ○ Analytical (ANA) vs. Fourier (FFT) field offset calculations;
- Diffusion options:
  ○ Monte Carlo (MC) diffusion vs. deterministic (DD) diffusion of spins.

These will all be described in detail in the following sections. Most combinations of options can be permuted, with a few exceptions, namely (1) the FFT method can only be applied in discretized space; (2) the custom geometry option can only be used with the FFT method. Representing the toolbox this way allows us to create a short but comprehensive naming scheme that can describe each method.Figure 1details how these names are generated and how to understand them.

**Fig. 2.**
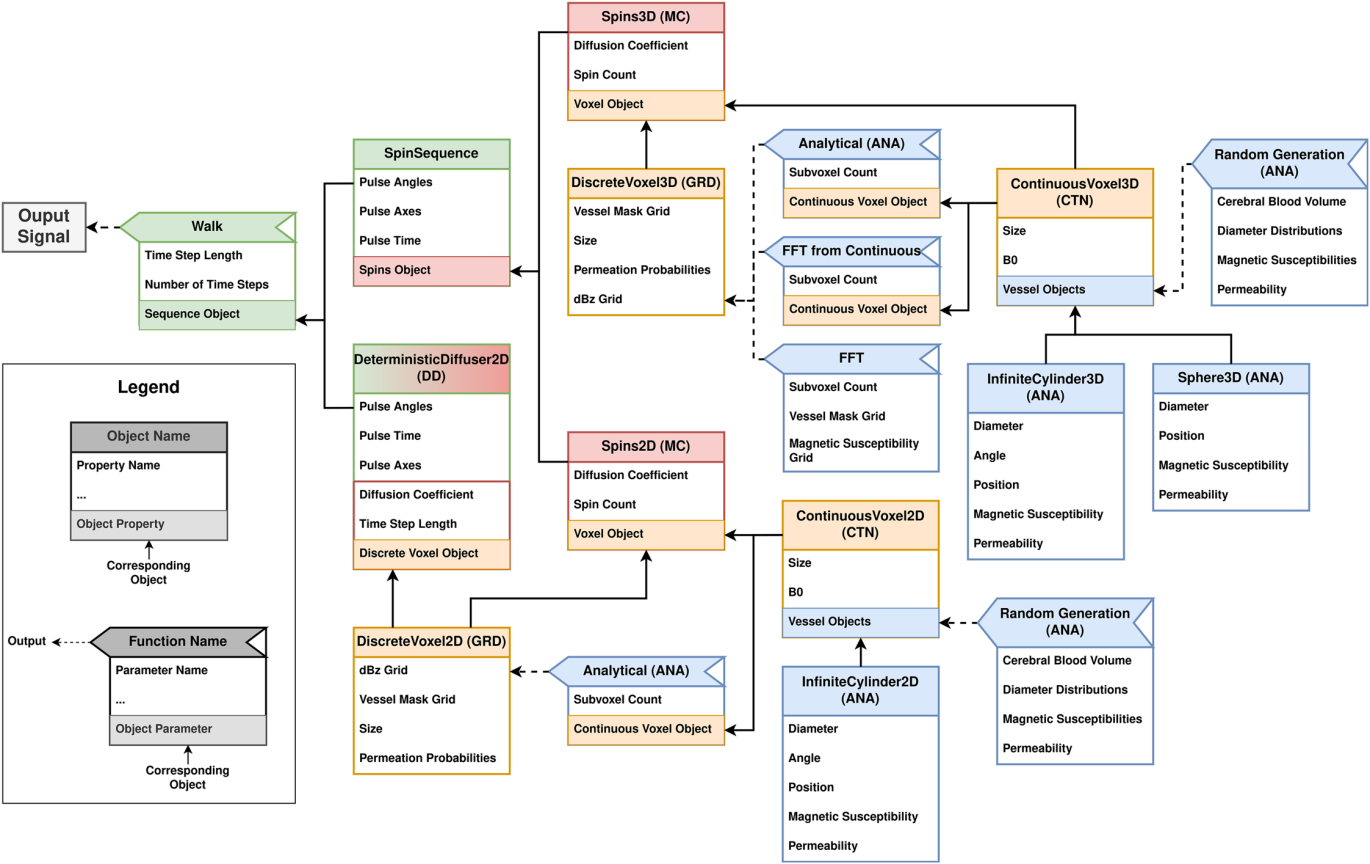
Module flow chart for the BOLDsωimsuite package. All currently possible simulation pipelines are shown for the case of a simple BOLD signal simulation. Each box represents either a class object or a member function, as shown in the legend. In each box are listed the attributes or parameters that are required or can be interacted with. The color of each object/method relates to the simulation component it represents, as shown inFigure 1. For example, the ContinuousVoxel3D object is yellow, meaning that it relates to the Geometry component, whereas the Spins2D object is red, meaning that it relates to the Diffusion Scheme component. Here, the DeterministcDiffuser2D object is an exception (colored red and green), as it takes care of both the Diffusion Scheme and Signal Calculation components. A single path connecting the output signal on the left back toward the far right of the diagram can be used to identify the necessary objects and methods to generate the Python script that will run the desired simulation.

Using the BOLDsωimsuite package, simulations are run in Python scripts built by the user.Figure 2shows a more in-depth view of the internal structure of the package, including functions and objects involved in a BOLD signal simulation. Depending on the simulation method desired, a specific subset of objects and functions are required to add all the necessary simulation components. Each of these objects or methods has attributes or parameters that need to be defined. These may be simple inputs or could be other objects (which need to be defined themselves). Presenting the toolbox this way provides a better understanding of how each method can be implemented and can be used as a preliminary guide for generating working simulation scripts.

## 3 Theory

All symbols and acronyms relevant to the theoretical description are listed inTable 1.

**Table 1.** List of symbols

Symbol	Definition
*α*	Flip angle (radian)
*B*_0_	Main magnetic field (tesla)
CBV	Cerebral blood volume (fraction of voxel volume)
*D*	Diffusion coefficient (mm ^2^ /s)
D	Diffusion kernel matrix used in the deterministic-diffusion method
∆B _z_	Magnetic field offset (T)
∆χ	Susceptibility difference between intra-cylinder (or intra-sphere) and extra-cylinder (or extra-sphere) spaces
γ	Gyromagnetic ratio (radian/s/T)
∆ω	Magnetic resonance frequency offset (radian)
Δt	Diffusion step size (s)
*k*_x_ , *k*_y_ , *k*_z_	k-space coordinates
N	Number of subvoxels (grid points)
N _spins_	Number of water spins used in Monte Carlo diffusion
N _steps_	Number of diffusion steps used in Monte Carlo diffusion
r	Magnitude of the vector perpendicular from the cylinder axis to the proton location
*R*	Vessel radius (mm)
R	MR relaxation matrix used in the deterministic-diffusion method
Φ	Angle between the vector r and the transverse component of *B*_0_
σ	Standard deviation of Gaussian diffusion kernel
W	Width of voxel (mm)
θ	Polar angle, also the angle between the *B*_0_ and the cylinder axis
*T*_1_	Longitudinal relaxation time (s). *R*_1_ = 1/ *T*_1_
*T*_2_	Transverse relaxation time (s). *R*_2_ = 1/ *T*_2_
*T*_2_ ’	Refocusable component of T2 (s). *R*_2_ ’ = 1/ *T*_2_ ’

First, we will clarify the basic concepts involved in the description of forward modeling as follows:

- Magnitude and phase: The MRI signal is complex and can be defined by its magnitude (the absolute value of the summation of the magnetic moments of all spins in a given voxel) as well as its phase (the rotational angle of this total magnetic moment) at the time of sampling, TE;
- A gradient-echo (GE) sequence is used to sample the free-induction decay, which follows the dephasing of spins, with the signal acquired at the TE exhibiting mainly*T*_2_* weighting;
- A spin-echo (SE) sequence includes a refocusing pulse (of any refocusing angle), in which the refocused signal is recorded with a*T*_2_weighting (nominal); the refocused (rephased) signal is produced at the TE, but an SE sequence in a broader sense can include a train of refocusing pulses, each producing a series of*T*_2_-weighted signals.
- The extravascular (EV) BOLD signal arises from the magnetic dipoles surrounding blood vessels (predominantly paramagnetic dipoles), which lead to enhanced*T*_2_* dephasing and MRI signal intensity decrease in a manner consistent with the blood properties and blood-vessel geometry;
- The intravascular (IV) BOLD signal arises from*T*_2_* decay associated with intravascular deoxyhemoglobin, but may also contain*T*_2_* decay contributions due to EV dephasing from neighboring blood vessels
- Grid element vs. voxel: a voxel is the smallest tissue volume that is capturable separately by the MRI technique, and is determined fundamentally by the image acquisition; in contrast, in the context of this paper, each voxel can be further divided into sub-voxels (or grid points) to simulate the underlying tissue microstructure; for instance, a typical fMRI voxel is 3 mm isotropic, which, for the purpose of gridded forward modeling, can be subdivided into 1,000 x 1,000 x 1,000 grid points.

### 3.1 Geometry

The definition of the simulation geometry requires three choices: number of dimensions, spatial discretization, and perturber geometry.

#### 3.1.1 Dimensionality

The choice between 2D (Berman et al., 2018;Miller & Jezzard, 2008;Ogawa et al., 1993;Pannetier et al., 2014) and 3D (Klassen & Menon, 2007;Martindale et al., 2008) simulations have major impacts on the compatible simulation pipeline and the geometry representation.

3D simulations operate in a cubic volume, while 2D simulations operate on a square plane, both called voxels (3D voxels and 2D voxels). A 3D voxel allows for more flexibility in the simulated geometry as it better represents real vasculature. However, 2D simulations are less computationally demanding and can provide equally accurate results to their 3D counterpart when the added flexibility is not required (Berman et al., 2023).

#### 3.1.2 Continuous and discrete space simulations

Simulations can be performed either on a continuous or discrete space. Continuous-space simulations offer greater spatial accuracy through the use of analytically defined perturbers and efficient sampling of the field offset. They also tend to be less memory intensive since they do not require large arrays that store spatially varying properties of the voxel (e.g., field offsets).

On the other hand, discretizing space allows for much more flexible perturber geometries (e.g., branching vessels) but incurs a tradeoff between spatial accuracy and computational efficiency. Discrete space simulations calculate the magnetic field offset over the entire voxel, with equally-spaced sampling, thereby forming grids (with*N*elements on each side, where*N*is defined by the user).

#### 3.1.3 Perturber definition and generation

BOLDsωimsuite can simulate three main classes of perturbers: infinite cylinders, spheres, and custom-defined perturber masks. The first two are defined entirely analytically and are generated using very few parameters, as will be described in the next section. They can be either defined by the user or generated randomly using parameters. Currently, the toolbox offers the functionality of generating randomly oriented cylinders and spheres in continuous space. The custom-defined perturber masks can accommodate any perturber geometry such as vascular anatomical networks (VANs). These require complete discretized masks, which must be generated externally. Sample perturber configurations are shown inFigure 3.

**Fig. 3.**
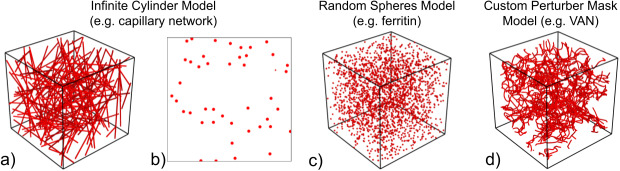
Depictions of sample voxels. Infinite-cylinder models can be constructed in 3D (a) or 2D (b). Microsphere and empirically determined microvascular (VAN) models can be constructed in 3c and d, respectively.

##### 3.1.3.1 Infinite cylinders

Infinite cylinders are a commonly used blood vessel model but are limited by their lack of curvature and branching. As a result, they have a varying degree of accuracy regarding the representation of realistic vasculature.

3D infinite cylinders are defined by their diameter, position, orientation, magnetic susceptibility, and water permeation probability (permeability). Both the position and orientation can be randomly generated, while all other parameters must be input manually. The method employed to randomly generate these parameters provides a uniformly distributed CBV (Martindale et al., 2008).

In a 2D simulation, infinite cylinders are always perpendicular to the voxel plane. To achieve a field offset distribution that is comparable to randomly oriented 3D infinite cylinders, each cylinder is assigned its own randomly oriented magnetic field direction (Miller & Jezzard, 2008). Each infinite cylinder is defined by its diameter, position, magnetic field orientation, magnetic susceptibility, and permeation probability. Both the position and magnetic field orientation can be randomly generated, while all other parameters must be input manually.

##### 3.1.3.2 Spheres

The spherical perturbers are not meant to represent vasculature, but can instead be used for a variety of other perturbers for more unconventional simulations such as red blood cells, ferritin (Brammerloh et al., 2021), or even polystyrene microspheres used in BOLD phantoms (Bieri & Scheffler, 2007). These perturbers are only implemented for 3D simulations. Spherical perturbers are defined by their diameter, position, magnetic susceptibility, and permeation probability. Like infinite cylinders, their position can be randomly generated while all other parameters must be input manually. Unlike cylinders, however, they do not have an orientation.

##### 3.1.3.3 Custom-defined perturber masks

Custom-defined perturber masks are only available for 3D simulations, and they rely on user-generated perturber geometry. Although this makes them the most flexible perturber, generating the perturber mask usually requires significant work outside the toolbox. For example, VANs can be generated using such methods as 2-photon microscopy angiographic imaging (X. Cheng et al., 2019;Gagnon et al., 2015).

### 3.2 B _0_ field offset calculation

The field offset in the simulation can be calculated in two distinct ways, either analytically or by FFT-based convolution.

#### 3.2.1 Analytical

Analytically defined perturbers allow for exact calculation of the magnetic field offset and can be used either in continuous or discrete space simulations. The magnetic field offset of infinite cylinder vessels is defined analytically as follows for the intravascular (IV) and extravascular (EV) spaces, based on (Ogawa et al., 1993),

$$\Delta B_{z}=\{\begin{array}{c} B_{0}\frac{\Delta \chi }{6}(3cos^{2}\left(\theta \right)-1),\;\;\;\;\;\;\;\;\;\;\;\;\;\;\;\;\;\;inside\;cylinder\\ B_{0}\frac{\Delta \chi }{2}{\left(\frac{R}{r}\right)}^{2}cos\left(2\varphi \right)sin^{2}\left(\theta \right),\;\;\;\;\;\;\;\;\;\;\;\;\;\;\;\;outside\;cylinder \end{array}$$ (1)

Where in both cases,*B*_0_is the applied magnetic field strength, and Δχ is the magnetic susceptibility difference between the intra- and extravascular spaces (in SI units).*R*is the cylinder radius, and*r*represents the distance between the cylinder’s axis and the spin position spanned by the vector**r**, θ represents the angle between the magnetic field direction and the cylinder axis, and φ is the angle between the vector**r**and the projection of*B*_0_onto the plane orthogonal to the cylinder axis.

The field offset associated with spherical perturbers is modeled as (Boxerman, Hamberg, et al., 1995)

$$\Delta B_{z}=\{\begin{array}{c} 0,\;\;\;\;\;\;\;\;\;\;\;\;\;\;\;\;\;\;\;\;\;\;\;\;\;\;inside\;sphere\\ B_{0}\frac{\Delta \chi }{3}{\left(\frac{R}{r}\right)}^{3}\left(3cos^{2}\left(\theta \right)-1\right),\;\;\;\;\;\;\;\;\;outside\;sphere \end{array}$$ (2)

where R is the sphere radius, r represents the distance between the sphere’s origin and the spin position, and θ represents the angle between*B*_0_and**r**.

#### 3.2.2 Fourier-based convolution

The magnetic field offset of a magnetization distribution can also be calculated through the discrete Fourier transform (Marques & Bowtell, 2005,^2008^). The local dipoles of the field perturbations can be calculated in the Fourier domain and then Fourier-transformed back to the spatial domain (Deville et al., 1979). Our implementation is based on the work byY.-C. N. Cheng et al. (2009), where the 3D B_Z_is defined as

$$B_{z}(\vec{r})={ℱ}^{-1}\{ℱ\{\chi (\vec{r})\}\;\cdot \;\{ℱ\{G_{z,3D}(\vec{r})\}\}B_{0}$$ (3)

The kernel*G*is first calculated in the spatial domain as (Y.-C. N. Cheng et al., 2009),

$$G_{z,3D}(\vec{r})\equiv \frac{1}{4\pi }\;\cdot \;\frac{3z^{2}-r^{2}}{r^{5}}$$ (4)

*G*_z,3D_is set to zero at*r*= 0. One clear advantage of the Fourier convolution method is its independence from analytical equations. Thus, the perturber layout and shape are arbitrary and can contain any geometry, including in vivo mapped VANs. However, the field offsets generated through Fourier convolutions are inherently discretized and thus have limited spatial resolution. Further, they are susceptible to boundary effects and wrap-around artifacts. To prevent these wrap-around artifacts, it is usually recommended to add zero padding to the arrays used in Fourier transforms (and inverse Fourier transforms). The amount of zero padding is set by the user, whereby selecting to have no padding will have the effect of mirroring the voxel at its edge boundaries (due to the periodicity of the discrete Fourier representation). This can be desired to emulate the presence of similarly dense vascular networks around the voxel, with the downside that this surrounding is just a mirrored version of the voxel. If*χ*(*r*), which fills the simulation voxel, is N elements wide along each dimension, and G_z,3D_is M elements wide on each dimension, then the padded voxel must be N+M-1 wide to prevent wrap-around.

### 3.3 Diffusion

Simulating diffusion effects using the BOLDsωimsuite can be done in two different ways, either with the gold standard Monte Carlo approach or using the convolution-based deterministic-diffusion method.

#### 3.3.1 Monte Carlo diffusion

Monte Carlo diffusion uses randomly positioned particles that move in the voxel through a random walk. Since the discovery of the effect of diffusion on BOLD contrast (Ogawa et al., 1993), there have been several efforts to standardize the implementation of Monte Carlo simulations (Boxerman, Bandettini, et al., 1995;Fieremans & Lee, 2018;Martindale et al., 2008), as well as to predict the effect of neurovascular coupling using forward modelling (Chen et al., 2018). In our implementation, consistent with these other works, each step length is randomly sampled from a normal distribution with a standard deviation of$\sqrt{2D\Delta t},$where D is the diffusion coefficient andΔtis the time step length. Note that the particles’ positions are always computed in continuous space, regardless of whether the simulation geometry is defined in continuous or discrete space. This allows for diffusion steps that are much smaller than the grid elements to cumulatively take effect. Voxel boundary conditions are imposed on the particles to model the behavior of diffusing water molecules. First, the voxel boundaries are periodic, such that any particle exiting the voxel on one end reappears on the other (and moved inward by the remaining diffusion distance). This is done to ensure that diffusion is not restricted by the voxel boundaries but also that particles do not leave the voxel’s volume.

##### 3.3.1.1 Perturber permeability

Another boundary is formed by the permeability of the perturbers. When a particle step crosses a perturber’s wall, it has a chance to successfully permeate the wall, given by the perturber’s “permeation probability”. If the permeation is successful, the particle moves to the new position, otherwise, a new step is randomly generated until one is found that does not cross the wall. Many simulations use either fully permeable or fully impermeable vessels, but the Monte Carlo diffusion implemented in BOLDsωimsuite allows for partially permeable perturbers (with a permeation probability between 0 and 1). This allows the simulation of various health conditions. For instance, healthy vasculature is often modeled as impermeable since the exchange rate of water is relatively long (~500 ms) (St.Lawrence et al., 2012). Blood-brain barrier breakdown, resulting in increased vessel permeability, occurs in many neurological disorders, for example, Alzheimer’s disease (Sweeney et al., 2018), tumors (Arvanitis et al., 2019), ischemic stroke (Kassner & Merali, 2015), and mild traumatic brain injury (Veksler et al., 2020). Furthermore, the simulations may not be restricted to just brain fMRI. Organs, such as the liver and kidneys, have much higher vascular permeability (van Hinsbergh, 2017).

At each time step, the particles accumulate a spin dephasing due to the magnetic field offset. This can be calculated from the*B*_0_offset using the Larmor equation. In the case of continuous space simulations, the dephasing is calculated for each spin at each timestep. For discrete space simulations, the*B*_0_offset is pre-calculated on the entire grid, and spin dephasing is sampled from the grid.

#### 3.3.2 Deterministic diffusion

The deterministic diffusion method represents diffusion as a smoothing process of convolving spin magnetizations with a Gaussian kernel, as diffusion of an ensemble of spins is Gaussian distributed (Bandettini & Wong, 1995). As such, it requires a discrete-space voxel, so that spin magnetization is defined at each point on the grid, rather than by individual diffusing spins. The magnetization at time point j is then given by

$${\text{M}}_{j\;}=\{\begin{array}{c} ({\text{M}}_{j-1}\;\cdot \;\text{R})*\text{D},\;\;\;\;j>0\\ 1,\;\;\;\;\;\;\;\;\;\;\;j\;=\;0 \end{array}$$ (5)

Where j is the time-step index, and**R**represents the*T*_2_relaxation process.**D**is the diffusion kernel. We implemented deterministic diffusion for 2D simulations using options of Gaussian and modified Bessel kernels. The Bessel kernel was first introduced (Pannetier et al., 2014) as an improvement over the 2D Gaussian kernel, as proposed earlier byLindeberg (1990). Elements of the Gaussian diffusion kernel are defined as

$$D_{k}\;=\;\frac{\Delta \text{x}}{\sigma \sqrt{2\pi }}exp\left(\frac{x_{k}^{\;2}+y_{k}^{\;2}}{2\sigma^{2}}\right)$$ (6)

Elements of the Bessel kernel are defined as

$$D_{k}=exp(-{\left(\sigma /\Delta x\right)}^{2})I_{k-N_{hw}}{\left(\sigma /\Delta x\right)}^{2}$$ (7)

where N_hw_is the number of samples at half of the kernel width,*k*is the sample index in the diffusion kernel width direction, and*I*_k_-*N*_hw_is the modified Bessel function of the first kind (order*k*-*N*_hw_).

Boundary conditions in the convolution are addressed by padding the voxel with itself, such that spins that hit the voxel boundary are wrapped to the other side. This enables the deterministic diffusion process to closely emulate the behavior of Monte Carlo diffusion.

##### 3.3.2.1 Perturber permeability

In the deterministic diffusion framework, perturbers are defined as fully permeable or impermeable. By default, the kernel convolution does not interact with perturber boundaries, which corresponds to fully permeable perturbers. It is optional to include a correction at each step which emulates impermeable perturber behavior by effectively returning any magnetization that crossed tissue boundaries back to the original tissue (Pannetier et al., 2014). Currently, no intermediate levels of permeability are implemented.

### 3.4 Signal calculation

The MR signal is generated from the net transverse magnetization,*M_xy_*, which is calculated throughout the simulation. Longitudinal magnetization,*M_z_*, is also calculated. To calculate*M_xy_*at each step, the change in the precessional frequency at position*p*is determined with

$$\Delta \omega (\vec{p})=\gamma \Delta B_{z}(\vec{p})$$ (8)

And the additional phase accumulated at each time step due to this frequency shift is given by

$$\Delta \Phi (\vec{p})=\Delta \omega (\vec{p})\;\cdot \;\Delta t$$ (9)

Thus, the transverse MR signal at time step*j*is defined as

$$M_{xy,j}(\vec{p})=M_{xy,j-1}(\vec{p})e^{i\Delta \Phi (\vec{p})}e^{-\Delta \text{t}/T_{2}(\vec{p})}$$ (10)

where the first exponential is the precession due to the field offset, and the second is the*T*_2_decay with a tissue-specific transverse relaxation time,*T*_2_(*p*). The BOLD signal is, in turn, computed as the magnitude of the sum of transverse magnetization.

$$S_{j}\;=\;\left|\sum_{i=1}^{N_{spins}}M_{xy,j}({\vec{p}}_{i})\right|$$ (11)

where*N_spins_*is the number of spins (or number of grid elements for deterministic diffusion).*M_z_*at each time step is governed by

$$M_{z,j}\;(\vec{p})=\;M_{z,j-1}(\vec{p})\left[e^{-\Delta t/T_{1}(\vec{p})}-1\right]$$ (12)

Where*T*_1_(*p*) is a tissue-specific longitudinal relaxation time. Note that both*T*_1_and*T*_2_can be user defined. If they are not defined, both decays are assumed to be negligible relative to*T*_2_’ decay or on the timescale considered and do not contribute to the resulting signal.

RF pulses are implemented by specifying the pulse axis in polar coordinates. For example, an RF pulse along the x-axis is specified by the orientation vector [π/2, 0], while a pulse along the y-axis is given by [π/2, π/2], where the first angle represents the polar angle, relative to the z-axis, and the second angle represents the azimuthal angle, relative to the +x-direction. Each pulse also requires a flip angle*α*(in radians) and the time step at which it is applied. Each pulse is applied to the total magnetization vector M_xyz_, and occurs at the specified time step just before M_xy_and M_z_are calculated.

## 4 Applications

### 4.1 Example: simulating a spin-echo BOLD signal

**Fig. 4.**
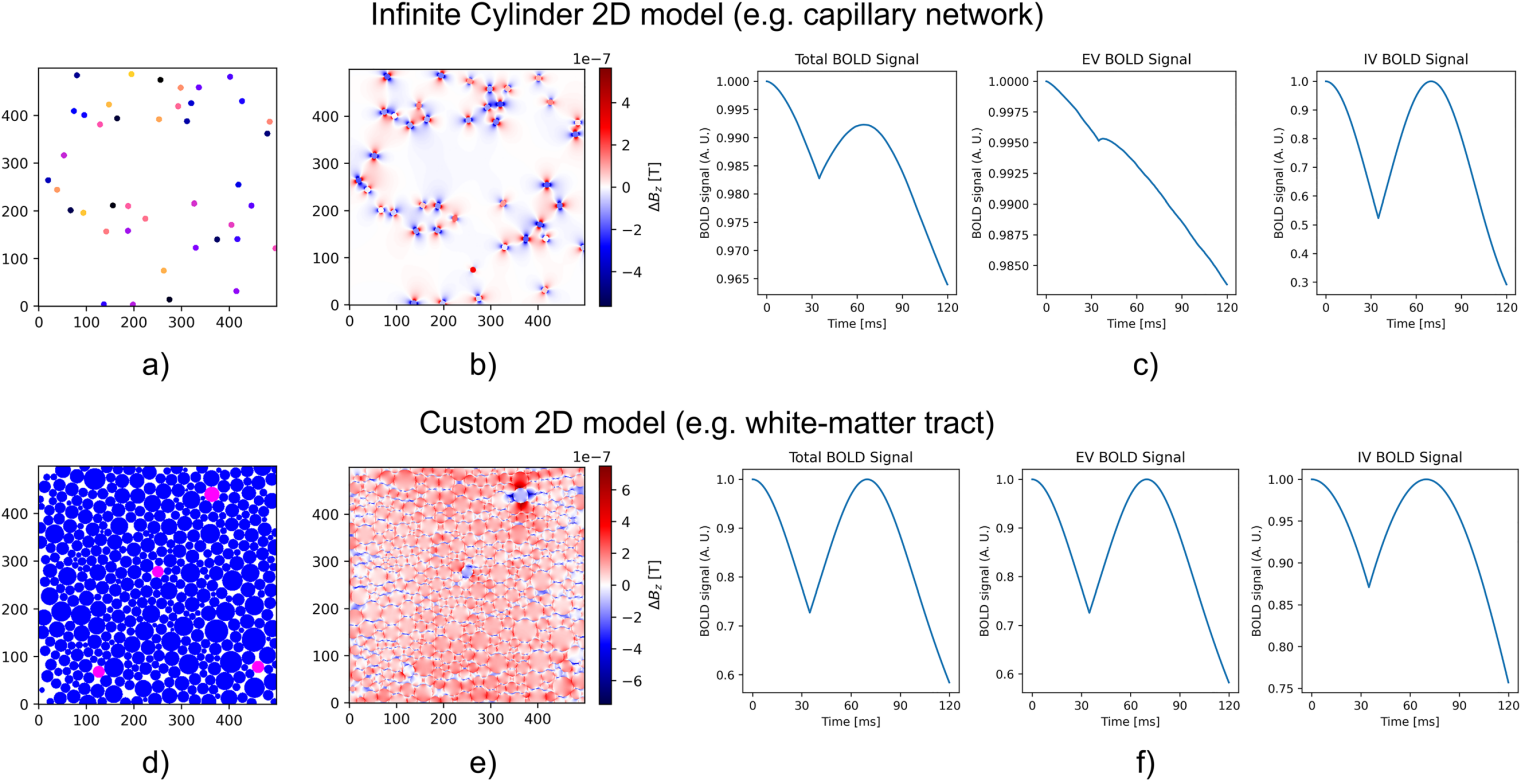
Sample simulated voxel configurations (2D) and corresponding spin-echo BOLD signals. (a) A network of infinite cylinders with random*B*_0_orientations is illustrated in a 2D voxel, in which the colors encode different perturbers. (b) The resultant ∆B_z_map, in which blue and red indicate the magnetic dipoles surrounding the cylindrical vessels. The axes indicate physical size in arbitrary units. (c) The corresponding BOLD time courses for a spin-echo sequence (TE = 70 ms) for the total BOLD signal as well as its intravascular (IV) and extravascular (EV) constituents. (d) A 2D voxel with densely packed axons (blue) and 1% CBV randomly oriented infinite cylinders (pink), with the circles representing axonal or blood vessel cross-sections. (e) The resultant ∆B_z_map, in which*B*_0_is at a 90^o^angle to the fibers in the upward direction. (f) The spin-echo BOLD signals corresponding to this white matter voxel.

Shown inFigure 4are two examples of BOLD signal time courses generated using the 2D Monte Carlo setting of BOLDsωimsuite.Figure 4aillustrates a network of infinite cylinders with random orientations (*θ*), which is typical of cortical grey matter. Vascular radius was set to be 1 μm in this example, CBV to be 0.02 and ADC to be 0.001 mm^2^/s. The resultant ∆B_z_map is shown inFigure 4b, and the corresponding BOLD time courses for a spin-echo (SE) sequence (TE = 70 ms) are shown inFigure 4cfor the total BOLD signal as well as its intravascular (IV) and extravascular (EV) constituents. We assumed Δχ of blood vessels as the intra-extravascular space difference (deoxygenated blood being paramagnetic), taken to be 0.3 ppm (cgs) based on past work (Spees et al., 2001). The signal undergoes gradient-echo*T*_2_* decay up to 35 ms, at which point the refocusing pulse is applied. InFigure 4d, we show the spatial map of a population of densely packed cylinders, such as those found in a white matter bundle (Denk et al., 2011;Fieremans & Lee, 2018;Sukstanskii & Yablonskiy, 2014;Wharton & Bowtell, 2012). Currently, these white matter fiber layouts can be reconstructed in the toolbox from user-defined inputs; in this case, these were generated by the AxonPacking package (Mingasson et al., 2017). The circles represent axonal cross-sections, defined to range from 0.4 to 3.5 μm in diameter, and*B*_0_is assumed to be at a 90^o^angle to the fibers. This bundle of largely parallel cylinders is interspersed with a few randomly oriented blood vessels. We set Δχ of the axons as the intra-extra-myelin susceptibility difference (myelin being diamagnetic), taken to be -0.15 ppm (cgs) in this example based on the range reported previously (Lee et al., 2012;Liu et al., 2011). Likewise, the spin-echo BOLD signals corresponding to this white matter voxel are shown inFigure 4f.

### 4.2 BOLD signal modeling based on vascular networks

**Fig. 5.**
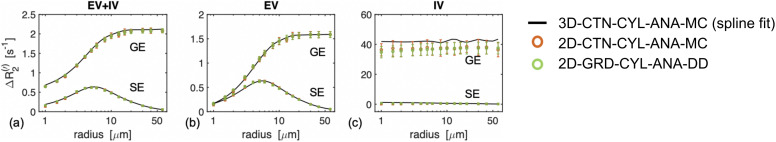
Comparison of Boxerman-style plots of Δ*R*_2_and Δ*R*_2_’ showing similarities and differences across simulation approaches. Δ*R*_2_^(’)^represents the transverse relaxation attributable solely to the susceptibility-induced field perturbations. The signal is split into three subplots: (a) the total signal, (b) the extravascular signal, and (c) the intravascular signal. The gradient-echo (GE) and spin-echo (SE) related relaxation rates were determined using three different simulation methods, namely 3D-CTN-CYL-ANA-MC, 2D-CTN-CYL-ANA-MC and 2D-GRD-CYL-ANA-DD (Refer toFig. 1for method naming breakdown). Figure adapted from Berman et al. (2023).

An obvious application for this toolbox is in BOLD signal modelling (Boxerman, Hamberg, et al., 1995;Kennan et al., 1994). Boxerman et al. (2023) simulated the GE and SE BOLD signals as a function of perturber radius, as well as the IV contribution. As was shown in our previous work, the implementation of a large set of simulation approaches within the toolbox enabled a direct comparison of the different simulation approaches. As an example,Figure 5ashows the dependence of the BOLD signal relaxation rates on the vascular radius, demonstrating broad agreement among the 3D-CTN-CYL-ANA-MC, 2D-CTN-CYL-ANA-MC, and 2D-GRD-CYL-ANA-DD methods. However, upon closer examination of the IV component, greater differences were found across methods, especially in the case of the GE signal.

### 4.3 Quantitative fMRI: calibrated BOLD and vascular MRF

Forward modeling of the transverse signal decay due to vascular perturbers was integral to the development of vascular MR fingerprinting (MRvF) techniques (Christen et al., 2014), and is important for the continued development of calibrated BOLD. In the calibrated BOLD model, simulations of the BOLD effect have lent key insight into the microvascular and metabolic contributions to the BOLD signal (X. Cheng et al., 2019;Gagnon et al., 2015), and continue to enable ways to optimize microvascular (thus neuronal) specificity (Berman et al., 2018). In the MRvF method, estimates of blood oxygenation, volume, and radius are obtained by matching the measured decay to a dictionary of simulated decay curves, the simulated decay curves must be as representative of the experimental conditions as possible. In the original MRvF paper, the 2D infinite-cylinder model with deterministic diffusion was used to generate the dictionary, and contrast enhancement was used to minimize the effect of background macroscopic field inhomogeneities. Christen et al. found different levels of dictionary-matching reliability for the three parameters. Upon close inspection, we found that the dictionary generation process could be influenced by the simulation approach. In our recent work (Berman et al., 2023), we demonstrated that using MRvF dictionaries built from different simulation approaches resulted in different error rates in MRvF metrics, as shown inFigure 6. In this case, the ground-truth radius values were used in generating the simulated experimental signal (based on the 3D-CTN-CYL-ANA-MC approach). While the use of the same approach in dictionary generation resulted in fully accurate radius estimation results, in another approach that was examined, the use of a dictionary based on the 3D-GRD-CYL-FFT-MC approach resulted in large estimation errors for some vascular radii. Thus, the optimal generation of MRvF dictionaries requires further investigation.

**Fig. 6.**
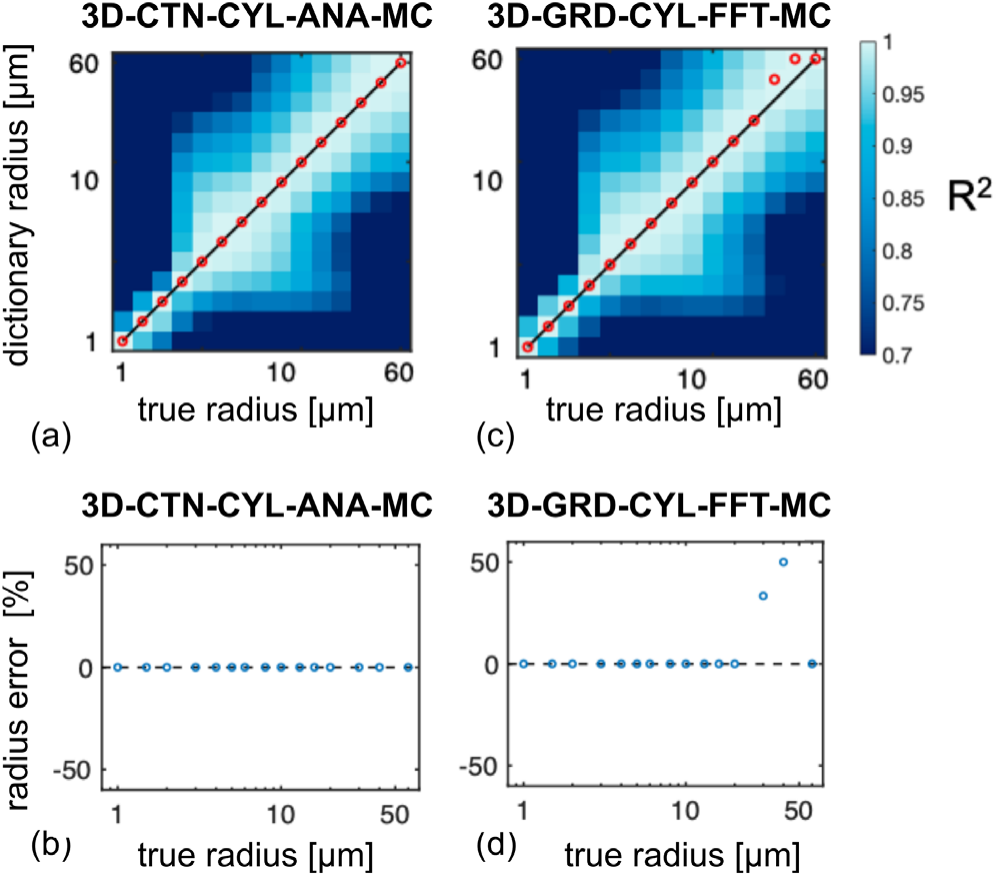
The accuracy of vascular MRF (MRvF) varies by simulation method. The coefficient of variation (R^2^) contour plots (a, c) indicate the uniqueness of the dictionary match at each ground-truth radius. At higher radii, the use of dictionaries generated using the 3D-CTN-CYL-ANA-MC and 3D-GRD-CYL-FFT-MC methods resulted in different vessel radius estimates (b, d). Figure adapted fromBerman et al. (2023).

### 4.4 T _2_ *-weighted signal modelling of microspheres: red blood cells, contrast agents and iron deposition

Given challenges in constructing tissue phantoms containing microvasculature, microspheres, which are easier to distribute evenly in a phantom medium, were suggested as a surrogate for microvascular networks (Scheffler et al., 2018). Our simulation work (Chausse, Berman, Polimeni, et al., 2021), in which we included SE, GE, and asymmetric SE (ASE) related transverse relaxation rates (*R*_2_’), where*R*_2_’ = 1/*T*_2_’, confirmed that microspheres do, indeed, behave like micro-cylinders in the context of BOLD contrast, as shown inFigure 7.

**Fig. 7.**
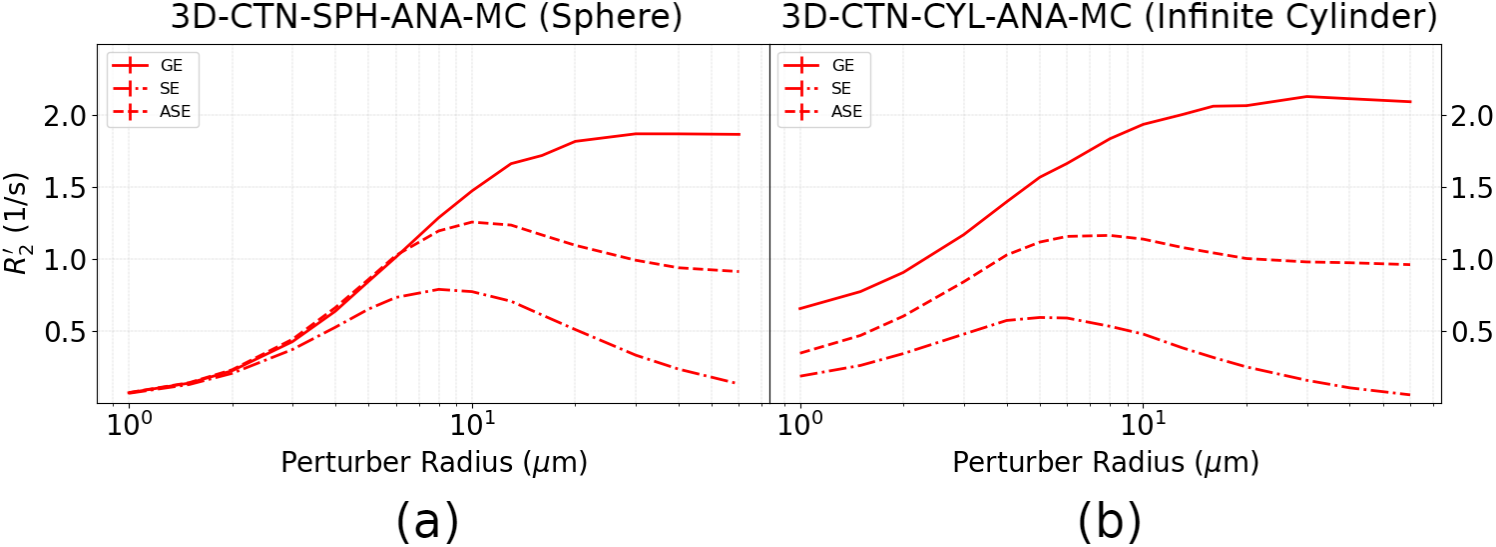
Comparison of Boxerman-style plots for micro-cylinder and microsphere-based voxel models, showing the dependence of transverse relaxation (*R*_2_’) on perturber radius. (a) EV R2’ based on a voxel of microspheres (note that microspheres are assumed to not have IV contributions); (b) the*R*_2_’ plot for infinite cylinders. In both cases, the susceptibility of all perturbers was assumed to be governed by an oxygenation level (Y) of 60%. The perturber radii are plotted on a log scale. Results are shown for gradient-echo (GE), spin-echo (SE), and asymmetric spin-echo (ASE) cases. Figure adapted fromChausse, Berman, Polimeni, et al. (2021).

In in vivo applications, in the work byBoxerman, Hamberg, et al. (1995), to simulate the effect of red blood cell (RBC) movement within a medium containing plasma and the contrast agent, RBCs were modeled as magnetized spherical perturbers. While this work found the flow of RBCs to contribute negligibly to the BOLD signal, the ability to model intravascular magnetized spheres easily extends to magnetized contrast particles (Pannetier et al., 2013). Such applications are not restricted to the brain, where contrast agents remain intravascular, but also to other organs, such as the liver, in which contrast agents can be modeled as uniformly distributed ferrite particles (Hernando et al., 2012;Yablonskiy & Haacke, 1994).

In addition to heme iron found in the RBCs, iron particles are present in vivo in three other main forms, ferritin, transferring, and neuromelanin. Ferritin, for instance, has a spherical shape that is typically about 12 nm in diameter (Mahroum et al., 2022), and when degraded forms hemosiderin. Ferritin accumulates in the aging process, while hemosiderin concentration is associated with brain disorders and hemorrhage. Different iron molecules induce different magnetic field offsets, and by modeling these molecules as spherical perturbers, one can estimate the concentration of these respective iron species based on*T*_2_-weighted MRI or*T*_2_MR relaxometry (Brammerloh et al., 2021).

## 5 Computational Considerations and Limitations

The BOLDsωimsuite toolbox offers multiple simulation approaches organized through combinations of independent modules (Fig. 1). To aid the choice of modules and approaches, we present the computational requirements (memory and time) associated with these choices and some general guidelines from the perspective of computational requirements. In the samples shown, simulation times are split into “setup” and “walk” portions, the former representing the time required to run the Geometry and B_0_Offset Calculation components and the latter representing the time to compute the Diffusion Scheme and Signal Calculation components. This separates the upfront time cost of building the system from the recurring cost of running additional time steps.

### 5.1 2D vs. 3D

In general, 3D simulations are more realistic and versatile; especially when VANs are required, 3D is the only option. However, in other situations, it is known that 2D vessels can accurately model voxels with randomly oriented vessels (Miller & Jezzard, 2008) and voxels with parallel vessels. Currently, there is no easy way to model more complex vascular configurations with 2D simulations such as non-cylindrical or non-random perturber distributions.

There are, however, certain cases for which a 2D voxel has clear advantages. For instance, the inclusion of randomly-oriented blood vessels in densely packed white matter fibres is simplified through the use of a 2D voxel. That is, the vessels and fibres can both be modelled as infinite cylinders, whereby random orientations of blood vessels can be accounted for conveniently by randomizing the orientation of the effective*B*_0_instead of re-orienting the vessels themselves. This avoids the complexities of orienting blood vessels in 3D without intersection with the densely packed surrounding axons. Moreover, computationally, 2D simulations are faster than 3D for equally large and complex systems (equal perturber count and grid size per dimension being the most important). Sample simulation timings are shown inFigure 8ato illustrate this. For continuous-space simulations, the 2D approach simplifies the analytical representation of vessels and the movement of spins, which can be seen as a small but significant gain in computational speed. Discrete-space simulations much more significantly benefit from this, and compared to their 3D counterpart, they have one fewer dimension (of size N) to discretize. Roughly speaking, this speeds up the discretization of continuous voxels and reduces the memory requirement by a factor of N, which can be observed inFigure 8b.

**Fig. 8.**
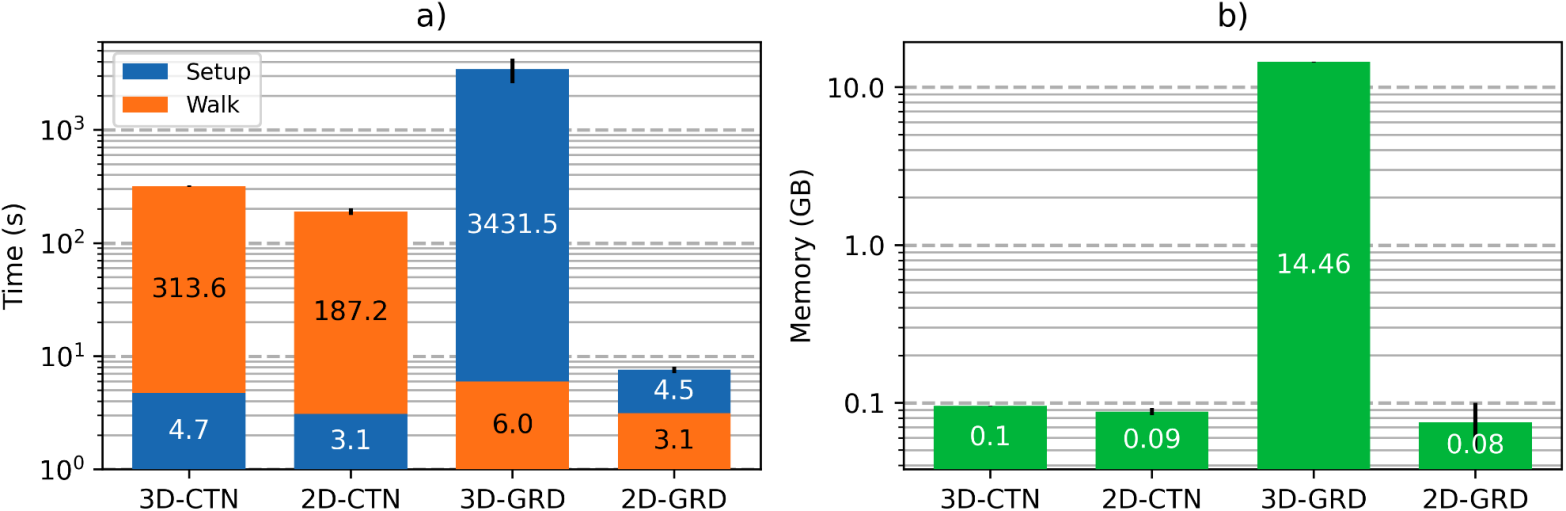
Computational times and memory requirements from sample simulations for 2D vs. 3D and continuous vs. discrete space. (a) Computational times. Voxel generation time (“Setup”) and Monte Carlo random walk time (“Walk”) are shown, with the lower of the two times displayed on the bottom portion of the bar due to the logarithmic y-axis. (b) Peak memory requirement during simulation time. The value (time or memory) associated with each component is labeled on the bars. Samples consist of an average of 10 simulations with randomly generated vasculature. All simulations were run for 600 time steps with 400 infinite cylinders at 2% CBV, an analytically calculated magnetic field offset and Monte Carlo diffusion (CYL-ANA-MC) with 40000 spins. For the discrete space simulations (GRD), the grid size was set to 500.

### 5.2 Choice of continuous vs. discrete space

For a meaningful comparison, this section only considers cases in which both continuous and discrete space representation are possible. Thus, since custom perturber geometries (e.g. VANs) are only possible with discrete-space simulations, they will not be included in this discussion.

One consideration for discretized simulations is sampling requirements. To allow for accurate discrete representations of diffusion and offset effects, the grid must sufficiently sample the perturber, as will also be discussed in the following subsection. We recommend that the smallest perturber be sampled with at least 6 grid elements across its diameter.

In nearly all cases, discrete-space simulations require significantly more memory than continuous space simulations, as the entire voxel space must be discretized and held in memory. On the other hand, for continuous simulations, the voxel’s spatial properties are calculated as needed using analytical equations, resulting in a smaller memory footprint. This is illustrated inFigure 8b, where we can see that the given discrete space simulation in 3D with N = 500 grid points is roughly 2 orders of magnitude more memory intensive than the continuous space equivalent.

Because of this, discrete-space 3D simulations should, in most cases, be considered when memory is not a limiting factor, when the discrete-space voxel will be reused or when the number of time steps is large. Regarding the reuse of a voxel, the discrete spatial properties can be saved to a file, thereby allowing the discretization step to be skipped in successive simulations of the same voxel. Although it still requires much more memory than a continuous-space simulation, any simulations done on the pre-computed discrete voxel will be much faster than the continuous-space equivalent. However, when voxels are not pre-computed or re-used, continuous-space simulations will often be faster, in addition to being more accurate. Discrete-space simulations can also be faster with a large number of time steps, as the magnetic field offset and EV/IV state of a spin is determined with a simple look-up of the grid rather than an analytical calculation. This is why the “Walk” time inFigure 8ais much shorter for the discrete-space methods. The exact point at which using discrete-space becomes faster than continuous-space for single simulations is dependent on the specific vascular network and method parameters, which therefore requires some testing by the user.

For voxels with analytically-defined field offsets (i.e., infinite cylinders or spheres in the present toolbox), discrete-space simulations can use either analytical or FFT methods for the field offset calculation. Generally, generating the discrete space voxel from the perturbers will be faster for the FFT method, and increasingly so when the number of perturbers is increased. However, the rest of the simulation procedure is identical for analytical and FFT in terms of computing requirements, as the voxels are then structurally identical. It is also important to note that using the recommended zero-padding in the FFT convolution will lead to higher memory requirements during the field offset calculation than the analytical method. Moreover, the FFT method is not exact and will introduce errors in the calculated magnetic field offsets. For these reasons, we recommend the use of analytically calculated voxels when possible.

### 5.3 Choice of Monte Carlo vs. deterministic diffusion

Deterministic diffusion simulations have the advantage of providing the same result every time, given the same parameters. Monte Carlo simulations by nature do not provide the same results unless provided with the same starting point (seed), and they also suffer from varying levels of signal noise, which can be difficult to adequately reduce.

**Fig. 9.**
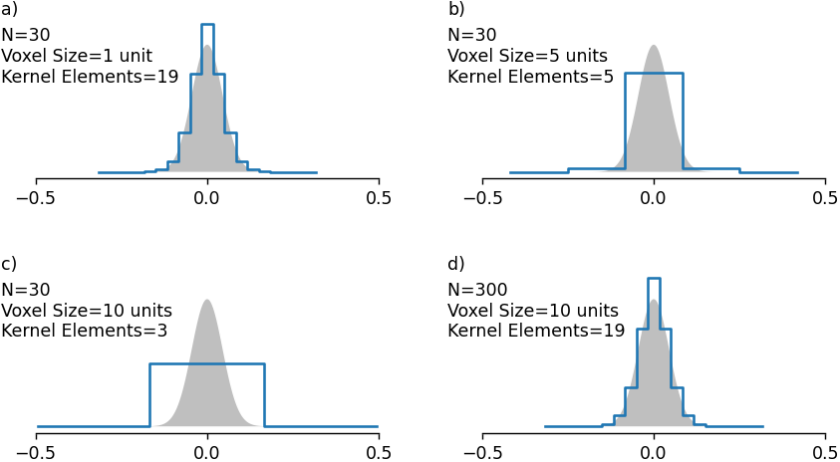
Diffusion kernel (blue line) vs. expected distribution (shaded area), showing over-discretization with increasing voxel size. (a-c) The diffusion kernel when increasing the voxel size without changing the grid-element count or the diffusion length. This results in a decreasing kernel width. (d) The diffusion kernel after the grid-element count in (c) is increased to match the size of each grid element in (a).

The computational requirements of deterministic methods can vary widely, as the voxel grid element count needs to be high enough to accurately represent the water diffusion distribution given by the diffusion kernel.Figure 9demonstrates this with an example. InFigure 9a, a diffusion kernel has been created for an N = 30 voxel of size 1 unit, resulting in a kernel containing 19 grid elements (represented by the blue line). This reasonably approximates the desired continuous function (shaded area).Figures 9band9cshow the same kernel creation process but for voxels of size 5 and 10 units, respectively (N remains the same). This could be the case for simulating voxels containing larger vasculature. We see here that due to the reduced grid resolution, the kernel contains fewer grid elements, no longer representing a reasonable approximation of the desired kernel function. This is problematic as it causes erroneous diffusion behaviour and leads to a lack of diffusion effect in the extreme case. The only solution for this is to increase N to maintain the same grid resolution, as shown inFigure 9d. However, doing so significantly increases both simulation time and memory requirements. It is important to note that this discretization problem is not nearly as impactful in Monte Carlo simulations, because our Monte Carlo simulations record the positions of diffusing spins in continuous space, irrespective of whether the voxel is discretized. As a result, though the field offset and vessel boundaries are discretized, the diffusion behaviour is not.

**Fig. 10.**
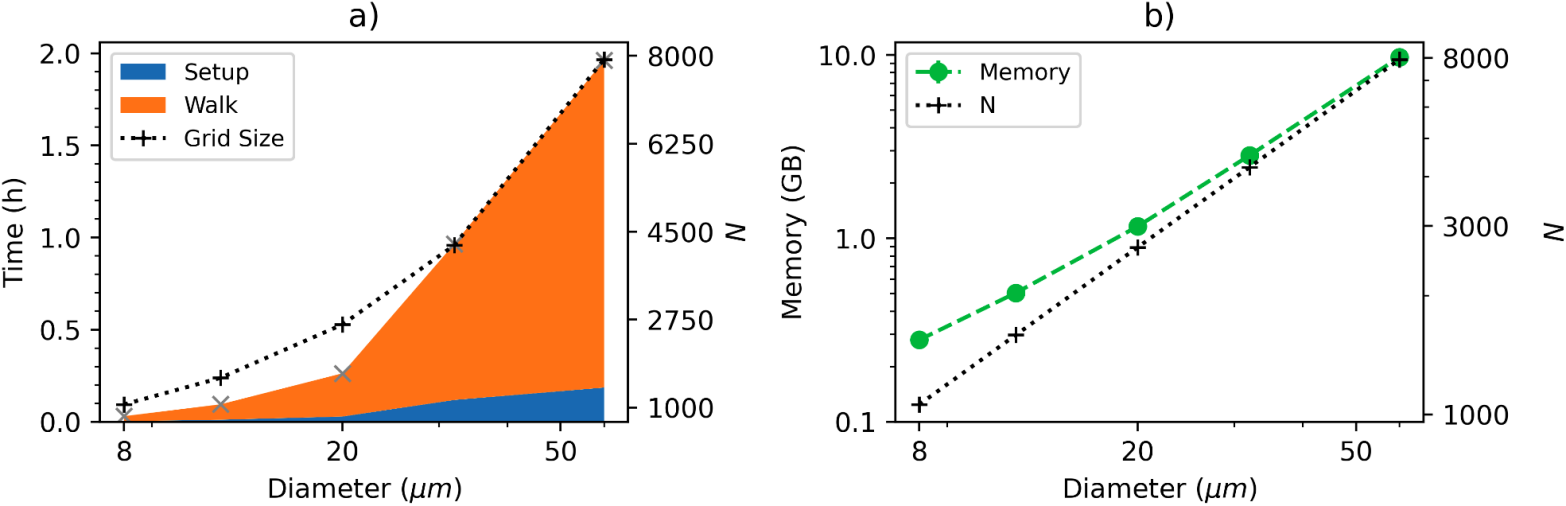
Computational times and memory requirements for sample simulations for varying diameters with the 2D deterministic method (2D-GRD-CYL-ANA-DD). (a) Computational times. Voxel generation time (Setup) and deterministic diffusion time (Walk) are shown. (b) Peak memory requirement during simulation time. All simulations were run for 600 time steps on voxels containing 400 cylinders at 2% CBV. The grid size per dimension is shown on the right y-axis of both figures and was calculated such that the diffusion kernel contained 11 elements.

The result of the increasing time and memory requirements with increasing vessel diameter for deterministic diffusion is shown inFigure 10. Here, sample simulations were run on identical sets of 10 voxels but with different vessel diameters. A constant kernel size of 11 elements was maintained for all the simulations, resulting in increasing grid sizes (as shown in both subplots).Figure 10ademonstrates this effect and the associated increases in computational times, which are several orders of magnitude greater than for the small vessel equivalent. In terms of memory,Figure 10bshows that the grid size and memory trends follow each other closely, as well as the large variations in the required memory, which can be on the order of 100 MB up to 10s of GB.

Lastly, for simulating 100% vascular permeability, 2D deterministic diffusion will in most cases be faster than the Monte Carlo equivalent. Full vessel permeability adds significant noise to the output signal in Monte Carlo simulations, requiring a significantly higher number of spins, but deterministic diffusion simulations do not have this issue and thus provide a noiseless signal without any changes to the parameters.

### 5.4 Comparison of BOLDsωimsuite with MrVox

Based on our understanding of the related publications (Pannetier et al., 2013,^2014^) and the publicly available code (https://github.com/NPann/MrVox2D), we perceive several differences between it and our toolbox. First, whereas MrVox implements only the 2D-GRD-CYL-FFT-DD method (and a simplification that uses the average field offset from only three*B*_0_directions) for randomly oriented cylinders, BOLDsωimsuite implements multiple combinations of methods, thus providing much higher flexibility. Available methods in BOLDsωimsuite include 2D-GRD-ANA-DD, 2D-ANA-MC (gridded and continuous), 3D-ANA-MC (gridded and continuous), 3D-GRD-FFT-DD, and 3D-GRD-FFT-MC. In fact, we recently compared the accuracy of the 3-B0 method from MrVox against other established methods (Berman et al., 2023), and show substantial differences. Moreover, BOLDsωimsuite provides the options of simulating spheres (for iron particle and blood-cell simulations) and receiving custom VANs as inputs. Third, BOLDsωimsuite is equipped with extensive documentation and tutorials that facilitate uptake by new users. Fourth, whereas MrVox is written in Matlab, BOLDsωimsuite is programmed entirely in Python and is therefore open-access. Fifth, BOLDsωimsuite is modular and easy to modify, leveraging the powerful version control of Github to facilitate open-source development by our users. This does not appear to be the case for the current version of MrVox.

### 5.5 Limitations and future developments

There are a number of limitations to the current toolbox. For instance, red blood cell can be modelled as 3D spheres in the current version of the toolbox. However, biconcave disks or ellipsoids or would more accurately represent red blood cells. These geometries could be added in future versions. Given that 2D simulations are substantially faster and less memory-demanding than 3D simulations, a 2D version of the randomly distributed sphere model (for modeling red blood cells or iron distributions) could be investigated. Moreover, while the current toolbox accommodates simple pulses sequences, it does not offer a straightforward way to simulate time-varying gradient fields that are typically encountered in image-encoding or diffusion-encoding. While it is possible to manually add a magnetic field gradient to the spins in Monte Carlo diffusion (as detailed in Lesson 6 in the toolbox tutorial document), future versions could incorporate a streamlined implementation of this feature.

## 6 Conclusions

In this paper, we presented a Python-based software toolbox, referred to as BOLDsωimsuite, that contains a suite of BOLD-signal forward modelling functionalities to suit modern computing hardware and infrastructures. This software suite, due to its comprehensiveness and flexibility, can facilitate reproducible science in the domain of quantitative MRI.

## Data Availability

The toolbox can be accessed by following the instructions on the package’s public GitHub:https://github.com/jacobchausse/BOLDswimsuite.
